# Re-examination of the Chinese record of *Opisthotropis maculosa* (Squamata, Natricidae), resulting in the first national record of *O. haihaensis* and description of a new species

**DOI:** 10.3897/zookeys.913.48622

**Published:** 2020-02-19

**Authors:** Jian Wang, Zhi-Tong Lyu, Zhao-Chi Zeng, Chao-Yu Lin, Jian-Huan Yang, Truong Quang Nguyen, Minh D. Le, Thomas Ziegler, Ying-Yong Wang

**Affiliations:** 1 State Key Laboratory of Biocontrol/The Museum of Biology, School of Life Sciences, Sun Yat-sen University, Guangzhou 510275, China Sun Yat-sen University Guangzhou China; 2 Department of Zoology, Graduate School of Science, Kyoto University, Kyoto 606-8502, Japan Kyoto University Kyoto Japan; 3 Kadoorie Conservation China, Kadoorie Farm and Botanic Garden, Lam Kam Road, Tai Po, Hong Kong, China Kadoorie Farm and Botanic Garden Hong Kong China; 4 Institute of Ecology and Biological Resources, Vietnam Academy of Science and Technology, 18 Hoang Quoc Viet, Cau Giay, Hanoi, Vietnam Institute of Ecology and Biological Resources, Vietnam Academy of Science and Technology Hanoi Vietnam; 5 Graduate University of Science and Technology, Vietnam Academy of Science and Technology, 18 Hoang Quoc Viet, Cau Giay, Hanoi, Vietnam Graduate University of Science and Technology Hanoi Vietnam; 6 Faculty of Environmental Sciences, VNU University of Science, Vietnam National University, Hanoi, 334 Nguyen Trai Road, Hanoi, Vietnam Vietnam National University Hanoi Vietnam; 7 VNU Central Institute for Natural Resources and Environmental Studies, Hanoi National University, 19 Le Thanh Tong, Hanoi, Vietnam Hanoi National University Hanoi Vietnam; 8 Department of Herpetology, American Museum of Natural History, Central Park West at 79th Street, New York, New York 10024, USA American Museum of Natural History New York United States of America; 9 AG Zoologischer Garten Köln, Riehler Strasse 173, D-50735 Cologne, Germany AG Zoologischer Garten Köln AG Zoologischer Garten Köln Germany; 10 Institute of Zoology, University of Cologne, Zülpicher Strasse 47b, D-50674 Cologne, Germany University of Cologne Cologne Germany

**Keywords:** New national record, *Opisthotropis hungtai* sp. nov., southern China, taxonomy

## Abstract

The taxonomic status of the previous record of *Opisthotropis maculosa* Stuart & Chuaynkern, 2007 from Guangdong and Guangxi, southern China, is revised based on the comparison of morphological and molecular data collected from the Chinese specimens and the holotype of *O. maculosa* from Thailand and *O. haihaensis* Ziegler, Pham, Nguyen, Nguyen, Wang, Wang, Stuart & Le, 2019 from Vietnam. Results reveal that the population from Shiwandashan Nature Reserve in southern Guangxi, China belongs to *O. haihaensis*, and represents the first national record for China; the populations from western Guangdong and southeastern Guangxi are described as a new species, *Opisthotropis hungtai***sp. nov.** We suggest that *O. maculosa* should be removed from the Chinese herpetofauna checklist. The new national record of *O. haihaensis* and the description of the new species bring the total number of *Opisthotropis* to 13 in China.

## Introduction

The genus *Opisthotropis* Günther, 1872 currently comprises 23 known species, and has spread widely throughout southern China and mainland of Southeast Asia, eastward to the Ryukyu Archipelago, southward to Sumatra and the Philippines (Ziegler et al. 2008; David et al. 2011, 2015; Yang et al. 2013; Teynié et al. 2014; Wang et al. 2017a, b). Currently, 12 species of the genus have been documented in China: *O. andersonii* (Boulenger, 1888), *O. cheni* Zhao, 1999, *O. guangxiensis* Zhao, Jiang & Huang, 1978, *O. jacobi* Angel & Bourret, 1933, *O. kuatunensis* Pope, 1928, *O. lateralis* Boulenger, 1903, *O. latouchii* (Bouleger, 1899), *O. laui* Yang, Sung & Chan, 2013, *O. maculosa* Stuart & Chuaynkern, 2007, *O. maxwelli* Boulenger, 1914, *O. shenzhenensis* Wang, Guo, Liu, Lyu, Wang, Luo, Sun & Zhang, 2017, and *O. zhaoermii* Ren, Wang, Jiang, Guo & Li, 2017. *O. balteata* (Cope, 1895) was recently transferred from the genus *Opisthotropis* to the genus *Trimerodytes* Cope, 1895 (Ren et al. 2019). Most species of the genus have been well recognized on the basis of phylogenetic and morphological analyses from the type series or topotypic specimens, especially in mainland China and northern Indochina (Ren et al. 2017; Wang et al. 2017; Ziegler et al. 2019). Nevertheless, the taxonomic statuses of several congeners remain unresolved, for instance, the records of *O. maculosa* from Guangdong and Guangxi in southern China (Yang et al. 2011).

**Figure 1.**
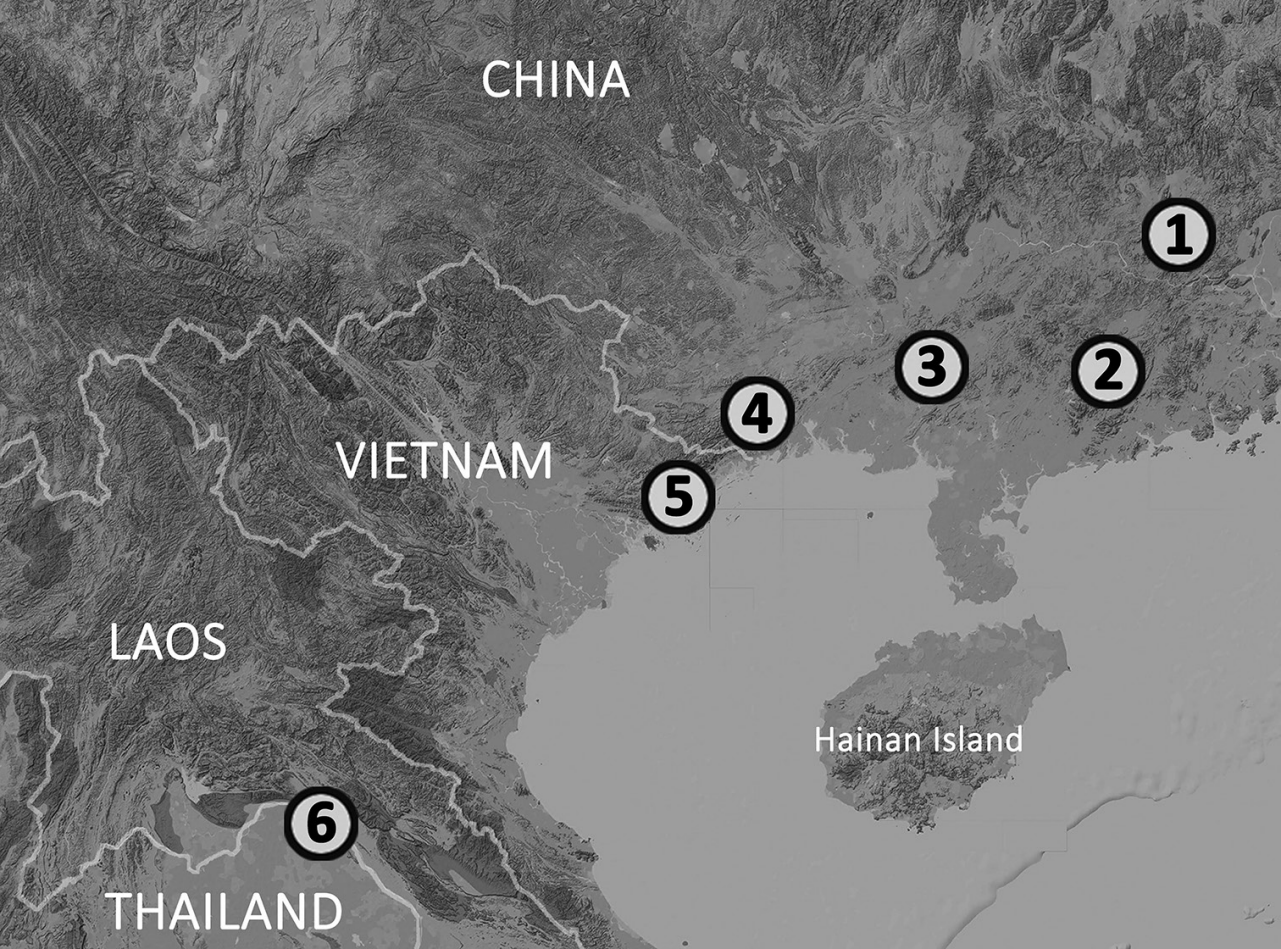
Collection localities of *Opisthotropis hungtai* sp. nov. (**1** the type locality, Heishiding Nature Reserve, Guangdong, China **2** Dawuling Forestry Station, Guangdong, China **3** Mt. Wuhuang, Guangxi, China), *O. haihaensis* (**4** Shiwandashan Nature Reserve, Guangxi, China **5** the forest near Tai Chi Village, Quang Ninh, Vietnam) and *O. maculosa* (**6** Phu Wua Wildlife Sanctuary, Nong Khai, Thailand), respectively.

*Opisthotropis maculosa* was originally described based on a single male specimen from northern Thailand (Fig. 1, site 6). Subsequently, it was reported based on morphological identifications in Guangdong (Fig. 1, sites 1, 2) and Guangxi (Fig. 1, sites 3, 4) in southern China (Yang et al. 2011), and northern Vietnam (Fig. 1, site 5) (Nguyen et al. 2018). Although some minor morphological differences among Chinese, Vietnamese and Thai populations were acknowledged in these publications, molecular data only became available recently, which lead to the resolution of the taxonomic statuses of the Vietnamese and Chinese records of *O. maculosa*. The Vietnamese record of *O. maculosa* was described as a distinct species, *O. haihaensis* Ziegler, Pham, Nguyen, Nguyen, Wang, Wang, Stuart & Le, 2019, by comparing the molecular and morphological data with the holotype of *O. maculosa* (Ziegler et al. 2019). In addition, their results also pointed out that the population of *O. maculosa* from Heishiding Nature Reserve in Guangdong, southern China may represent another distinct lineage.

In the present study, the *Opisthotropis* specimens from Guangdong and Guangxi, southern China previously recorded as *O. maculosa*, were re-examined using an integrative taxonomic approach, by combining results from both morphological and molecular analyses. In particular, morphological comparisons among the Chinese ‘*O. maculosa*’, the true *O. maculosa* from Thailand and the recently described *O. haihaensis* from Vietnam were undertaken in detail. The results demonstrate that the populations from southeastern Guangxi and western Guangdong represent a distinct taxon, which is described as a new species; the population from southern Guangxi is identified as *O. haihaensis*.

## Material and methods

### Morphometrics

Morphological examinations were performed on the holotype of *Opisthotropis haihaensis*, specimens reported as *O. maculosa* by Yang et al. (2011), and several other newly collected specimens. The collection information is given in the taxonomy accounts below. All specimens were fixed in 10 % buffered formalin and later transferred to 70 % ethanol for preservation, and deposited in the Museum of Biology, Sun Yat-sen University (**SYS**), Kadoorie Farm and Botanic Garden (**KFBG**), and Institute of Ecology and Biological Resources, Hanoi, Vietnam (**IEBR**).

Measurements followed Wang et al. (2017) and Ziegler et al. (2019) and were taken with digital calipers to the nearest 0.1 mm. These measurements were as follows:

**TL** total length (from tip of snout to tip of tail);

**SVL** snout-vent length (from tip of snout to posterior margin of cloacal plate);

**TaL** tail length (from posterior margin of cloacal plate to tip of tail).

Scalation features and their abbreviations are as follows: preoculars (**PrO**); postoculars (**PtO**); supralabials (**SPL**); infralabials (**IFL**); temporals (**TMP**); ventral scales (**V**); subcaudals (**SC**); dorsal scale rows (**DSR**) were counted at one head length behind head, at midbody, and at one head length before vent, respectively. Bilateral scale counts were given as left/right.

Maxillary teeth counts (**MT**) were determined by subequal teeth or sockets on right upper maxilla, and sex was determined by dissection or by the presence/absence of everted hemipenis.

### Phylogenetic analyses

The mitochondrial cytochrome *b* (CYTB) gene was used for molecular analyses. Two new samples from Mt. Wuhuang, southeastern Guangxi and Shiwandashan Nature Reserve, southwestern Guangxi, were included in our study. DNA extraction, PCR amplification and sequencing followed the protocol employed by Wang et al. (2017). In addition, 31 *Opisthotropis* and two outgroup sequences (following Ziegler et al. 2019) were attained from GenBank for the phylogenetic analysis (Table 1).

**Table 1.** Localities, voucher information, and GenBank numbers for all samples used in this study.

ID	*Opisthotropis* Species	Voucher No.	Collection locality	Genbank No.
1	*Opisthotropis hungtai* sp. nov.	SYS r000538 (Paratype)	CHINA: Guangxi: Mt. Wuhuang	MN890018
2	*Opisthotropis hungtai* sp. nov.	SYS r000946 (Holotype)	CHINA: Guangdong: Heishiding Nature Reserve	KY594748
3	*O. andersonii*	SYS r001423 (Topotype)	CHINA: Hongkong: Tai Tam	KY594730
4	*O. andersonii*	SYS r001424 (Topotype)	CHINA: Hongkong: Tai Mo Shan	KY594731
5	*O. cheni*	YBU071040 (Topotype)	CHINA: Hunan: Mangshan Nature Reserve	GQ281779
6	*O. cheni*	SYS r001422	CHINA: Guangdong: Shimentai Nature Reserve	KY594741
7	*O. daovantieni*	ROM FS39306	VIETNAM	MK941140
8	*O. durandi*	NCSM 80739	VIETNAM	MK941137
9	*O. guangxiensis*	GP746	CHINA: Guangxi	GQ281776
10	*O. haihaensis*	IEBR A.2016.34 (Holotype)	VIETNAM: Quang Ninh: Hai Ha District	MK991139
11	*O. haihaensis*	SYS r000537	CHINA: Guangxi: Shiwandashan Nature Reserve	MN890017
12	*O. jacobi*	IEBR 4329	VIETNAM: Vinh Phuc: Tam Dao	MG545601
13	*O. jacobi*	ZFMK 100818	VIETNAM: Vinh Phuc: Tam Dao	MG545602
14	*O. kuatunensis*	SYS r001008	CHINA: Fujian: Shanghang County	KY594746
15	*O. kuatunensis*	SYS r001081	CHINA: Guangdong: Mt. Wutong	KY594747
16	*O. laui*	SYS r001161	CHINA: Guangdong: Shangchuan Island	KY594738
17	*O. laui*	SYS r001170	CHINA: Guangdong: Shangchuan Island	KY594739
18	*O. lateralis*	SYS r000951	CHINA: Guangdong: Heishiding Nature Reserve	KY594743
19	*O. lateralis*	SYS r001080	CHINA: Guangdong: Mt. Wutong	KY594744
20	*O. lateralis*	–	CHINA: Guangxi	GQ281782
21	*O. latouchii*	SYS r000670 (Topotype)	CHINA: Fujian: Mt. Wuyi	KY594742
22	*O. latouchii*	GP647	CHINA: Fujian	GQ281783
23	*O. maculosa*	FMNH 265798 (Holotype)	THAILAND: Nong Khai: Phu Wua Wildlife Sanctuary	MK991138
24	*O. maxwelli*	SYS r000841	CHINA: Guangdong: Nan’ao Island	KY594736
25	*O. maxwelli*	SYS r001053	CHINA: Fujian: Huboliao Nature Reserve	KY594737
26	*O. shenzhenensis*	SYS r001018 (Holotype)	CHINA: Guangdong: Mt. Wutong	KY594727
27	*O. shenzhenensis*	SYS r001021 (Paratype)	CHINA: Guangdong: Mt. Sanzhoutian	KY594728
28	*O. shenzhenensis*	SYS r001032 (Paratype)	CHINA: Guangdong: Mt. Tiantou	KY594729
29	*O. voquyi*	ZFMK 100819 (Paratype)	VIETNAM: Bac Giang: Tay Yen Tu Nature Reserve	MG451049
30	*O. voquyi*	ZFMK 100820 (Paratype)	VIETNAM: Bac Giang: Tay Yen Tu Nature Reserve	MG451050
31	*O. zhaoermii*	CIB109998 (Paratype)	CHINA: Hunan: Guzhang County	MG012799
32	*O. zhaoermii*	CIB109999 (Holotype)	CHINA: Hunan: Guzhang County	MG012800
33	*O. zhaoermii*	CIB110000 (Paratype)	CHINA: Hunan: Guzhang County	MG012801
**Outgroups**				
34	*Aspidura drummondhayi*	RS-M	SRI LANKA: Nuwara Eliya	KC347455
35	*Aspidura trachyprocta*	RS-134	SRI LANKA: Nuwara Eliya	KC347458

Amino acid sequences for the CYTB gene of all samples were first aligned using Clustal W with default parameters. After checking the alignment to make sure that there was no stop or error codon, the amino acid sequence dataset was transformed to a nucleotide sequence dataset. We then applied JModelTest v2.1.2 on the nucleotide sequence dataset under Akaike and Bayesian information criteria to determine the best-fit nucleotide substitution model. The dataset was analyzed using maximum likelihood (ML) in RaxmlGUI 1.3 (Silvestro and Michalak 2012) and Bayesian inference (BI) in MrBayes 3.2 (Ronquist et al. 2012) with the GTR + I + G model. For ML analysis, a bootstrap consensus tree inferred from 1000 replicates was used to represent the evolutionary history of the taxa analyzed. Branches reproduced in less than 50% of bootstrap replicates were collapsed. For BI analysis, two independent runs with four Markov Chain Monte Carlo simulations were performed for ten million iterations and sampled for every 1000^th^ iteration. The first 25% of samples were discarded as burn-in. Convergence of the Markov Chain Monte Carlo simulations was assessed using Tracer v.1.4 (http://tree.bio.ed.ac.uk/software/tracer/). We also calculated pairwise sequence divergence based on uncorrected *p*-distance using MEGA 6.06 (Tamura et al. 2013).

## Results

**Figure 2.**
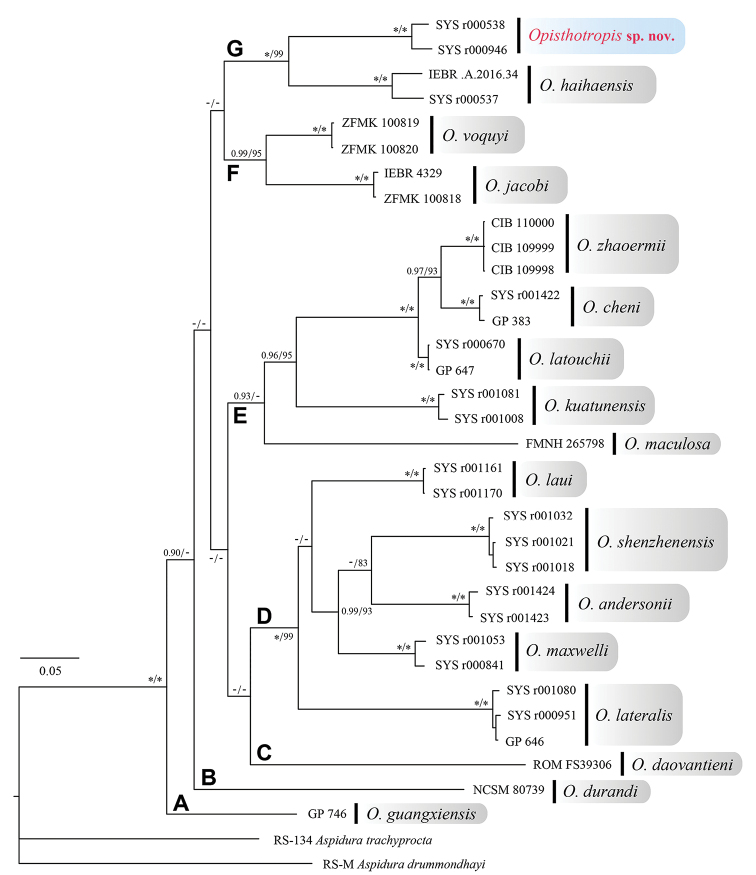
Bayesian Inference and Maximum Likelihood phylogenies. The Bayesian posterior probabilities (BPP) > 0.90 and the bootstrap supports for Maximum Likelihood analysis (BS) > 80 were retained.

The CYTB nucleotide sequence matrix contained 1059 characters without insertion-deletions. The MP and BI analyses produced essentially identical topologies, which were integrated in Fig. 2. Major nodes of the tree were sufficiently supported, with Bayesian posterior probabilities (BPP) > 0.90 and bootstrap supports (BS) for maximum likelihood analysis > 80. Uncorrected *p*-distances among *Opisthotropis* species based on the CYTB gene are shown in Table 2.

**Table 2.** Uncorrected *p*-distances among *Opisthotropis* species based on partial mitochondrial CYTB gene.

**Part 1**	**ID**	**1–2**	**3–4**	**5–6**	**7**	**8**	**9**	**10–11**	**12–13**	**14–15**
*Opisthotropis hungtai* sp. nov.	**1–2**	2.8								
*O. andersonii*	**3–4**	18.4–19.4	0.9							
*O. cheni*	**5–6**	20.3–20.8	20.4–21.0	0.5						
*O. daovantieni*	**7**	22.5–23.0	18.8	21.1–21.2	–					
*O. durandi*	**8**	20.3	20.3–20.5	21.4–21.6	20.8	–				
*O. guangxiensis*	**9**	18.0–18.6	16.7	18.9–19.3	21.0	17.9	–			
*O. haihaensis*	**10–11**	14.7–15.3	18.2–18.7	18.1–18.4	21.6–22.3	19.8–20.1	16.5–16.6	4.6		
*O. jacobi*	**12–13**	15.9–16.7	18.2–19.0	16.9–17.3	19.3–19.5	18.0–18.2	15.4–15.6	15.8–16.3	0.4	
*O. kuatunensis*	**14–15**	18.1–19.2	19.0–19.5	15.9–16.5	20.7–21.5	20.2	18.3–18.4	18.9–19.7	17.3–17.6	1.1
*O. laui*	**16–17**	17.5–19.4	13.6–14.2	19.3–19.6	21.4–21.5	20.9–21.1	18.9–19.0	17.6–18.8	17.8–18.0	19.2–19.6
*O. lateralis*	**18–20**	20.7–22.2	16.5–16.7	17.9–18.3	22.2–22.8	19.9–20.0	19.3–19.6	18.3–18.6	20.3–20.7	19.0–19.4
*O. latouchii*	**21–22**	19.3–19.5	18.6–19.0	5.3–5.4	20.1	20.3	17.9	17.7–18.6	16.9–17.0	15.6–16.0
*O. maculosa*	**23**	21.1	21.7–21.9	20.9	22.2	19.4	18.4	18.8–20.4	18.2–18.3	19.2–19.3
*O. maxwelli*	**24–25**	16.8–17.7	13.0–13.7	18.4–19.3	20.9–21.6	20.0–20.9	17.9–20.3	17.3–18.6	17.4–18.7	17.7–18.3
*O. shenzhenensis*	**26–28**	18.8–19.1	12.0–12.5	19.9–20.4	21.8–22.2	20.7–21.3	18.9–19.2	20.2–20.8	18.5–18.9	20.1–21.2
*O. voquyi*	**29–30**	17.0–17.3	16.2–16.5	16.3–16.8	19.7–19.9	17.6–17.7	14.9–15.0	15.0	10.9–11.5	18.0–18.2
*O. zhaoermii*	**31–33**	19.6–20.0	18.3–18.7	5.9	21.2	20.8	18.4	17.9–18.1	16.1	16.8–17.0
**Part 2**	**ID**	**16–17**	**18–20**	**21–22**	**23**	**24–25**	**26–28**	**29–30**	**31–33**	
*O. laui*	**16–17**	0.1								
*O. lateralis*	**18–20**	17.1–17.8	0–0.9							
*O. latouchii*	**21–22**	18.9–19.1	18.3–18.4	0						
*O. maculosa*	**23**	22.2–22.3	20.6–20.8	20.1	–					
*O. maxwelli*	**24–25**	12.8–13.4	15.0–16.1	18.0–18.5	20.6–20.9	1.5				
*O. shenzhenensis*	**26–28**	15.8–16.3	16.2–17.0	18.8–19.2	21.5–21.7	11.8–12.8	0–0.7			
*O. voquyi*	**29–30**	15.3–15.5	16.6–16.7	16.0–16.1	18.9–19.0	16.4–17.6	17.0–17.2	0.1		
*O. zhaoermii*	**31–33**	19.1–19.3	18.5–18.6	5.4	20.6	17.6–18.3	18.6–18.8	16.4–16.6	0	

In our phylogenetic tree, all samples of the genus *Opisthotropis* clustered in a monophyletic group with high nodal supports (BPP 1.00 and BS 100), and can be divided into seven clades, although the relationships among these clades were unresolved. *Opisthotropis daovantieni* Orlov, Darevsky & Murphy, 1998, *O. durandi* Teynié, Lottier, David, Nguyen & Vogel, 2014 and *O. guangxiensis* formed three monotypic clades, respectively. *Opisthotropis andersonii*, *O. lateralis*, *O. laui*, *O. maxwelli*, and *O. shenzhenensis* were grouped in clade D (BPP 1.00 and BS 99). Clade E (BPP 0.93) contained *O. cheni*, *O. kuatunensis*, *O. latouchii*, the true *O. maculosa*, and *O. zhaoermii*. The sister species *O. jacobi* and *O. voquyi* Ziegler, David, Ziegler, Pham, Nguyen & Le, 2018 constituted clade F (BPP 0.99 and BS 95).

Within clade G (BPP 1.00 and BS 99), the *Opisthotropis* sample (SYS r000537) from Shiwandashan Nature Reserve, southern Guangxi, was placed with the holotype of *O. haihaensis* from northeastern Vietnam, with high node support values (BPP 1.00 and BS 100) and moderate genetic distance (*p*-distance 4.6%). The detailed morphological examination suggests that they represent individuals of the same species. Thus, we herein revise the identification of the specimen as *O. haihaensis*, and report it as a new national record for China.

Besides, the *Opisthotropis* samples from Mt. Wuhuang, southeastern Guangxi and Heishiding Nature Reserve, western Guangdong, were reconstructed as a monophyletic clade with strong nodal supports (BPP 1.00 and BS 100) and small genetic distance (*p*-distance 2.8%). The populations should be considered as a distinct taxon, which is sister to *O. haihaensis*. These specimens show almost no morphological differences from those collected at Dawuling Forestry Station, western Guangdong, which is located in the same mountain belt as Heishiding Nature Reserve. Therefore, we describe these specimens as a new species, *Opisthotropis hungtai* sp. nov.

### Taxonomy accounts

#### Opisthotropis haihaensis

Taxon classificationAnimaliaSquamataNatricidae

Ziegler, Pham, Nguyen, Nguyen, Wang, Wang, Stuart & Le, 2019

F647EBFB-1331-588A-B4AF-3C13533B53D4

Figures 3 , 5A

##### Chresonymy.

*Opisthotropis maculosa* Stuart & Chuaynkern, 2007: Yang et al. (2011) (part); Nguyen et al. (2018).

##### Holotype.

IEBR A.2016.34 [Field No. QN 2016.91], adult female, from the forest near Tai Chi Village, Quang Son Commune, Hai Ha District, Quang Ninh Province, 950 m asl., Vietnam [exact locality and coordinates not provided owing to threat from collection for the pet trade (Ziegler et al. 2019)], collected by Cuong The Pham and Tan Van Nguyen on 9 May 2016.

**Figure 3.**
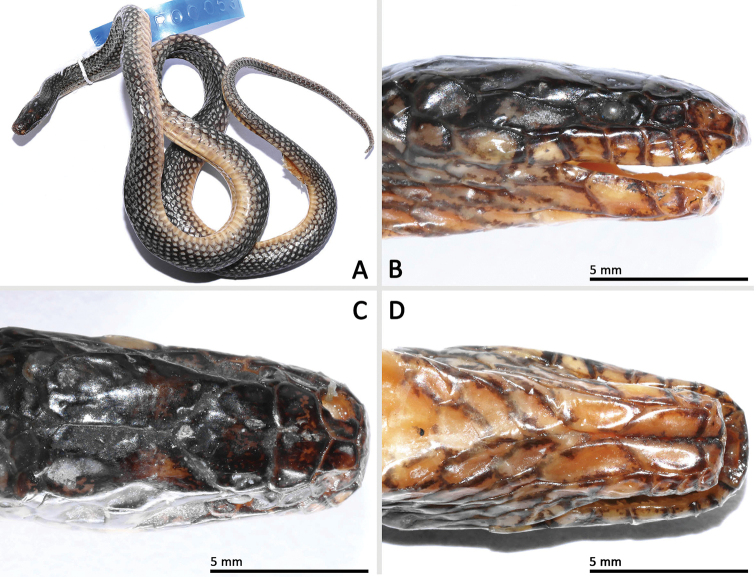
Morphological features of *Opisthotropis haihaensis* (SYS r000537) from Shiwandashan Nature Reserve, Guangxi, China. **A** Habitus view **B–D** close-up of head scales. Photos by Jian Wang.

##### Specimens examined (N = 1).

SYS a000537, adult female, collected by Qing Du and Jian-Huan Yang on 10 July 2009 from Shiwandashan Nature Reserve [exact coordinates not provided owing to threat from collection for the pet trade], Shangsi County, Qinzhou City, Guangxi Zhuang Autonomous Region, 493 m a.s.l., P.R. China.

##### Etymology.

According to the original description, the specific name “*haihaensis*” refers to the type locality of this species, Haiha District (Quang Ninh Province) in Vietnam. As this species is currently reported in China, we suggest its Chinese name “Hai He Hou Leng She (海河后棱蛇)”, derived from its scientific name.

##### Variation.

Measurements, scalation and body proportions of the two specimens are listed in Table 3. The specimen from China conforms to the holotype from Vietnam except for: (1) a shorter body size: snout-vent length 391.3 mm, tail length 108.9 mm (vs. SVL 396 mm, Tal 113 mm in the holotype); (2) a higher number of postoculars: PtO 2 (vs. PtO 1 in the holotype); (3) a lower number of maxillary teeth: MT 22 (vs. MT 24 in the holotype); (4) a lower number of ventrals: V 164+2 (vs. V 169+2 in the holotype); (5) a lower number of subcaudals: SC 75 (vs. SC 79 in the holotype); and (6) tail scales indistinctly keeled (vs. tail scales smooth in the holotype).

**Table 3.** Measurements, scale counts and body proportions of *Opisthotropis haihaensis* and *Opisthotropis hungtai* sp. nov.

**Species**	***O. haihaensis***		***Opisthotropis hungtai* sp. nov.**							
**No.**	IEBR A.2016.34	SYS r 000537	SYS r 000720	SYS r 001350	SYS r 001525	SYS r 001515	SYS r 000946	SYS r 000538	SYS r 002017	KFBG 2002.01
**Locality**	Haiha	Shiwandashan	Heishiding	Heishiding	Heishiding	Dawuling	Heishiding	Mt. Wuhuang	Dawuling	Dawuling
**Sex**	Female	Female	Female	Female	Female	Female	Male	Male	Male	Male
**TL**	509	500.2	511	470.2	393.2	435.9	501.2	464.3	366	483.5
**SVL**	396	391.3	431	383	312	337.9	401.6	343.6	318	373.1
**TaL**	113	108.9	80 (broken)	87.2	81.2	98	99.6	120.7	48 (broken)	110.4
**TaL/SVL**	0.22	0.22	broken tail	0.19	0.21	0.22	0.20	0.26	broken tail	0.23
**DSR**	15–15–15	15–15–15	15–15–15	15–15–15	15–15–15	15–15–15	15–15–15	15–15–15	15–15–15	15–15–15
**MT**	24	22	17	16	17	18	16	17	18	18
**PrO**	1/1	1/1	1/1	1/1	1/1	1/1	1/1	1/1	1/1	1/1
**PtO**	1/1	2/2	1/1	1/1	1/1	1/1	1/1	1/1	1/1	1/1
**SPL**	8 (3–2–3)	8 (3–2–3)	7 (3–2–2)	7 (3–2–2)	7 (3–2–2)	7 (3–2–2)	7 (3–2–2)	7 (3–2–2)	7 (3–2–2)	7 (3–2–2)
**IFL**	8/8	8/8	7/7	7/7	7/7	8/8	7/7	8/8	8/9	8/8
**TMP**	1+1/1+1	1+1/1+1	1+1/1+1	1+1/1+1	1+1/1+1	1+1/1+1	1+1/1+1	1+1/1+1	1+1/1+1	1+1/1+1
**V**	169	164	168	170	170	175	170	189	180	172
**SC**	79	75	56 (broken)	69	70	84	76	98	37 (broken)	83

##### Revision of original diagnosis.

*Opisthotropis haihaensis* is characterized by the combination of the following characters: (1) TL 500.2–509 mm in adult females, (2) tail relatively long, TaL/TL 0.22, (3) internasal not in contact with loreal, prefrontal not touching supraocular, frontal touching preocular, (4) one preocular, one or two postocular(s), (5) temporals 1+1, (6) supralabials eight, fourth and fifth in contact with eye, (6) 22–24 maxillary teeth, (7) anterior pair of chin shields longer than posterior pair; (8) ventrals 164–169 (+ 2 preventrals), (9) subcaudals 75–79, (9) nasal cleft pointing to the first supralabial, (10) body scales in 15–15–15 rows, (11) body scales smooth, tail scales smooth or indistinctly keeled, (12) chin shields yellow with brownish black mottling, and (13) body and tail dorsum dark, each with a light yellow spot per scale.

##### Coloration in life (SYS r000537).

Eye black; scales on dorsal surface of head glossy black with scattered yellow flecking; chin shields yellow with brownish black mottling; body and tail glossy black with iridescence above, with single yellow spot on each scale, yellow spots becoming larger on sides of body; ventrals yellow with brownish black lateral margins and scattered brown flecks; subcaudals yellow with brownish black anterior and lateral margins in both specimens.

##### Coloration in preservation (SYS r000537).

Ground color of upper head and body surface dark brown, that of venter yellowish-beige. Dorsal scales each with light blotch in the center. Dorsal tail scales likewise with light central blotches. Dorsal head surface in part with indistinct light mottling. Anterior supralabials with large light mottling. Infralabials, chin shields and smaller throat scales anterior to ventrals yellowish-beige with dark brown mottling per scale. Belly with few, scattered dark flecks on sides. Outermost edges of light ventrals brown. Ground color of subcaudals brown with transversally enlarged light blotches at each scale end.

##### Distribution and habits.

*Opisthotropis haihaensis* is currently known from its type locality, the forest near Tai Chi Village (ca 950 m a.s.l.), Quang Ninh, northern Vietnam, and Shiwandashan Nature Reserve (ca 500 m a.s.l.), southwestern Guangxi, southern China. The straight-line distance between the two localities is approximately 150 kilometers, indicating that the distribution area of this species is the mountain region on the border between China and Vietnam.

The holotype was found at night in a small rocky stream at 21:30h. The surrounding habitat was secondary evergreen forest consisting of small hardwoods, bamboo, and shrubs. The air temperature was 24–29 °C and the relative humidity was 65–88%. The holotype revealed to be an adult female, as dissection showed up to 16.5 mm long eggs and the oviducts were folded, indicating that eggs had already been laid (Ziegler et al. 2019). Besides, the other specimen, SYS r000537, was collected from a rocky stream (about 8 m wide and 0.3 m deep at the collecting site) running through well-preserved, dense deciduous forests. The collected individual was spotted swimming at night and swiftly hiding under stones when disturbed.

#### Opisthotropis hungtai sp. nov.

Taxon classificationAnimaliaSquamataNatricidae

5476E925-0F41-5D72-91AB-8EC4480C8DB9

http://zoobank.org/A0FC2C8F-866B-4D81-9237-1E42942711AA

Figures 4 , 5B , 6

##### Chresonymy.

*Opisthotropis maculosa* Stuart & Chuaynkern, 2007: Yang et al. (2011) (part); Wang et al. (2017a), Ren et al. (2019).

##### Holotype.

SYS r000946, adult male, collected by Jian Zhao on 2 September 2014 from Heishiding Nature Reserve [exact coordinates not provided owing to threat from collection for the pet trade, same as paratypes], Fengkai County, Zhaoqing City, Guangdong Province, 300 m a.s.l., P.R. China.

**Figure 4.**
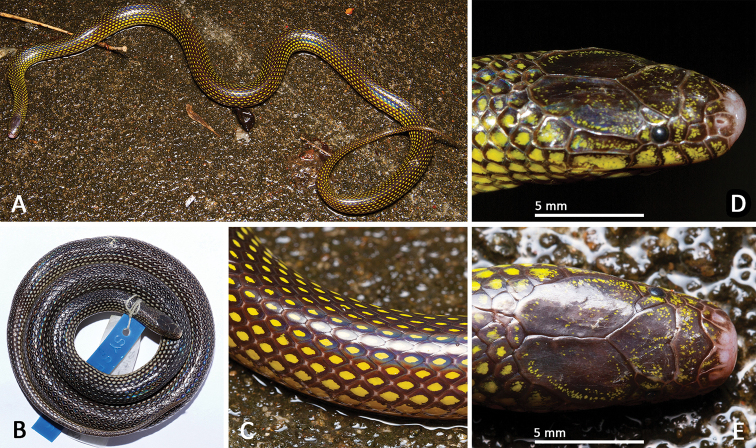
Morphological features of the adult male holotype SYS r00946 of *Opisthotropis hungtai* sp. nov. **A** Habitus view in life **B** habitus view in preservative **C** close-up of mid-dorsal body **D–E** close-up of head scales. Photos by Jian Zhao and Jian Wang.

##### Paratypes (N = 7).

Adult female SYS r001350 collected by Zhi-Tong Lyu on 15 August 2015, adult female SYS r000720 collected by Ying-Yong Wang on 28 June 2012, and adult female SYS r001525 collected by Zhi-Tong Lyu and Ying-Yong Wang on 1 July 2016, from the same locality as the holotype. Adult male KFBG 2002.01 collected by Zhi Xiao on 2 July 2002, adult male SYS r002017 collected by Jian Wang on 14 June 2018, and adult male SYS a001515 collected by Jian Wang on 8 July 2017, all from Dawuling Forestry Station, Xinyi City, Maoming City, Guangdong Province, ca 1150 m a.s.l., P.R. China. Adult male SYS a000538, collected by Qing Du and Runlin Li on 14 July 2009 from Mt. Wuhuang, Pubei County, Qinzhou City, Guangxi Zhuang Autonomous Region, ca 360 m a.s.l., P.R. China.

##### Etymology.

The species name “*hungtai*” refers to Professor Hung-Ta Chang (=Hong-Da Zhang, 张宏达), an outstanding botanist, who established the Tropical and Subtropical Forest Ecosystem Experimental Center in Heishiding Nature Reserve, promoting the development of ecological research in southern China. We suggest the English common name Hung-Ta Chang’s mountain Keelback and the Chinese name Zhang Shi Hou Leng She (张氏后棱蛇).

##### Diagnosis.

*Opisthotropis hungtai* sp. nov. is characterized by the following combination of characters: (1) TL 464.3–501.2 mm in adult males, 393.2–511 mm in females, (2) tail moderate, TaL/TL 0.20–0.26 in males, 0.19–0.22 in females, (3) internasal not in contact with loreal, prefrontal not touching supraocular, frontal touching preocular, (4) one preocular, one or two postocular(s), (5) temporals 1+1, (6) supralabials seven, the fourth and fifth in contact with eye; (6) maxillary teeth 16–18, (7) anterior pair of chin shields longer than or equal to posterior pair; (8) ventrals 170–189 (+ 2 preventrals) in males, 168–175 (+ 2 preventrals) in females, (9) subcaudals 76–98 in males, 69–84 in females, (9) nasal cleft pointing to the second supralabial, (10) body scale in 15–15–15 rows, (11) body scales smooth, tail scales smooth or indistinctly keeled, (12) chin shields yellow with brownish black mottling, and (13) body and tail dorsum dark, each with a light spot per scale.

##### Comparisons.

*Opisthotropis hungtai* sp. nov. is compared with *O. maculosa* and *O. haihaensis*, which share a very similar appearance. Measurements, scalation and body proportions of *O. haihaensis* and *Opisthotropis hungtai* sp. nov. are listed in Table 3.

*Opisthotropis hungtai* sp. nov. differs from *O. maculosa* by prefrontal not touching supraocular (vs. prefrontal touching supraocular in *O. maculosa*), by frontal touching preocular (vs. frontal not touching preocular in *O. maculosa*), by fourth and fifth supralabials in contact with eye (vs. fourth supralabial in contact with eye in *O. maculosa*), by anterior pair of chin shields longer than or equal to posterior pair (vs. anterior pair of chin shields shorter than posterior pair in *O. maculosa*), by a higher number of subcaudals, 76–98 in males (vs. 67 in the single male holotype of *O. maculosa*), and by chin shields yellow with brownish black mottling (vs. immaculate in *O. maculosa*).

**Figure 5.**
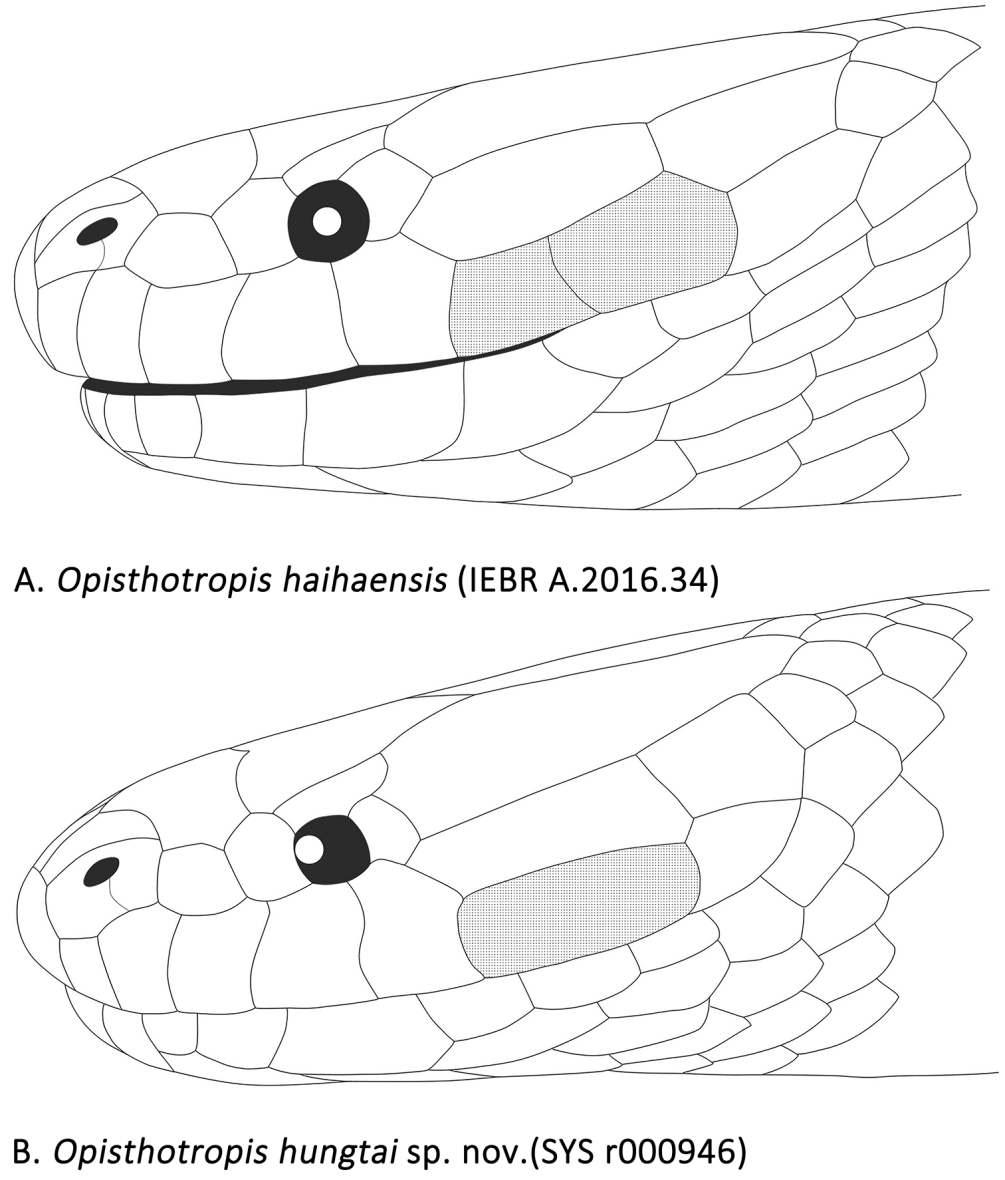
Comparisons of head scalation of *Opisthotropis haihaensis* and *Opisthotropis hungtai* sp. nov. Line illustration by Zhi-Tong Lyu.

*Opisthotropis hungtai* sp. nov. differs from *O. haihaensis* by having seven supralabials, the second last one significantly enlarged, narrow and long, significantly wider than high (vs. eight supralabials, the second last one slightly enlarged, slightly wider than high in *O. haihaensis*) (Fig. 5), and MT 16–18 (vs. MT 22–24 in *O. haihaensis*).

##### Description of holotype.

Body cylindrical, slender, round to oval in cross section; TL 501.2 mm (SVL 401.6 mm, TaL 99.6 mm); tail thin and pointed, TaL 20% of TL; head small, indistinct from neck; right upper maxilla with 16 subequal teeth or sockets, teeth small, curved, without diastema; rostral nearly flattened, small, slightly less than twice as broad as deep, barely visible from above; two internasals, crescent-shaped, in contact with each other medially behind the rostral, not in contact with loreal, posteriorly in contact with prefrontal; a single prefrontal, in contact with loreal and preocular laterally, with frontal posteriorly, not in contact with supraocular; a single frontal, hexagonal, in contact with supraocular laterally, with two parietals posteriorly; parietals large, in contact with each other medially; nasal directed dorsally, polygonal, in contact with first and second supralabials ventrally, with loreal and prefrontal posteriorly, with internasal dorsally, with rostral anteriorly; nostril horizontally oval, in the upper part of nasal; a short vertical cleft below the nostril and dividing nasal into anterior and posterior parts, pointing to middle of upper edge of second supralabial; a single loreal, trapezoid, not entering the orbit, in contact with second and third supralabials laterally; a single supraocular, much longer than wide, obliquely set; a single preocular, higher than wide, in contact with frontal; a single postocular; a single anterior temporal, significantly elongate, in broad contact with the elongated sixth supralabial; a single posterior temporal, pentagonal; supralabials 7/7, the sixth one significantly elongate, the last one much shorter than the adjacent preceding supralabial; fourth and fifth supralabials entering orbit; infralabials 7/7, the first one in contact with its fellow behind the mental; two pairs of chin shields; anterior chin shields larger, in contact with each other medially, and in contact with the first four infralabials on both sides; posterior chin shields smaller, in contact with each other; dorsal scales in 15–15–15 rows; dorsal scales of body smooth throughout; dorsal scales of tail weakly keeled; ventrals 170; cloacal plate divided; subcaudals 76, paired.

##### Coloration of holotype in life.

Eye black; scales on dorsal surface of head glossy dark brown with scattered yellow flecking; chin shields yellow with brownish black mottling at each margin; body and tail glossy dark brown with single yellow spot on each scale, yellow spots becoming larger on sides of body; ventrals yellow with brownish black lateral margins and few scattered brown flecks; subcaudals yellow with brownish black anterior and lateral margins.

##### Coloration of holotype in preservative.

Ground color of upper head and body surface dark brown (Fig. 4B), that of venter yellowish-beige. Dorsal scales each with light yellow blotch in the center. Dorsal blotches almost equal in size. Blotches becoming wider towards body sides; largest at outermost dorsal scale row, where the light blotches stretch towards the posterior scale end. Dorsal tail scales likewise with light central blotches. Dorsal head surface in part with indistinct light mottling that becomes more obvious on temporals. All supralabials with a light blotch. Infralabials, chin shields and smaller throat scales anterior to ventrals light yellow with brown mottling/blotches per scale. Belly with few, scattered dark flecks. Outermost edges of light ventrals brown. Ground color of subcaudals light yellow with black anterior and lateral margins.

##### Variations.

**Figure 6.**
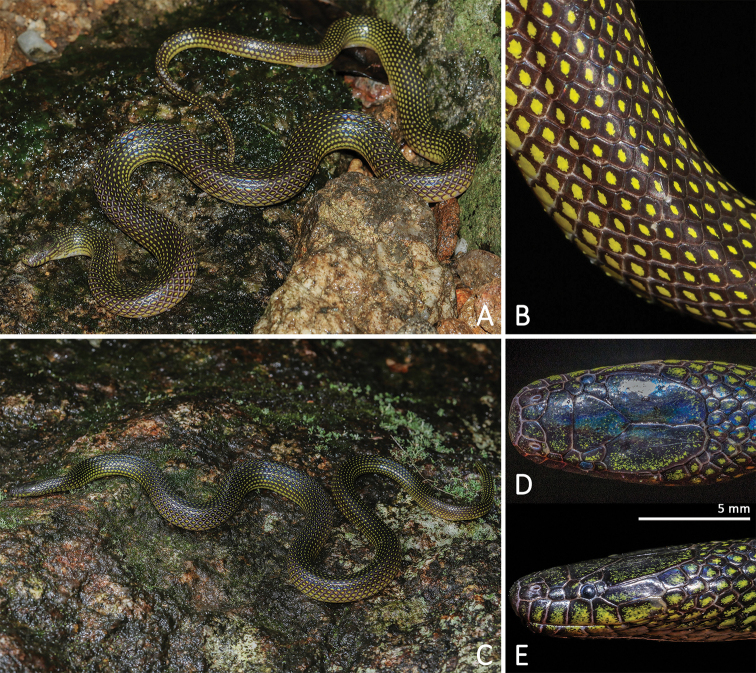
Morphological features of the adult female paratypes of *Opisthotropis hungtai* sp. nov. from Dawuling Forestry Station, Guangdong, China. **A, B** Habitus view and close-up of mid-dorsal body of SYS r001515 **C–E** habitus view and close-up of head scales of SYS r002017. Photos by Jian Wang.

Measurements, body proportions and scale counts are listed in Table 3. All paratype specimens are very similar to the holotype in appearance (Fig. 6) except: more maxillary teeth, ventrals and subcaudals, and relatively longer tail length in specimens KFBG 2002.01, SYS r001515, 2017 from Dawuling Forestry Station and SYS r000538 from Mt. Wuhuang; in the three female specimens from the same locality (Heishiding Nature Reserve) as the holotype, there are 17 maxillary teeth (vs. 16 maxillary teeth) and fewer subcaudals, 56 (broken tail) in SYS r000720, 69 in SYS r001350, 70 in SYS r001525 (vs. 76 in the male holotype).

##### Distribution and habits.

*Opisthotropis hungtai* sp. nov. is currently known from Heishiding Nature Reserve (ca 300 m a.s.l.) and Dawuling Forestry Station (ca 900 m a.s.l.) in western Guangdong, and Mt. Wuhuang (ca 500 m a.s.l.) in southeastern Guangxi.

The specimen from Mt. Wuhuang was collected in a rocky stream. Besides, specimens from Heishiding Nature Reserve were found in pelitic gutterways along the dirt path, and specimens from Dawuling Forestry Station were collected in a pelitic stream. The collection sites were all surrounded by well-preserved, dense deciduous forest.

## Discussion

As a representative snake group of the Oriental Realm, the mountain Keelback genus *Opisthotropis* receives more attention for its important role as an environmental indicator. Mountain Keelbacks are generally adapted to rocky forest streams (Wang et al. 2017a, b). However, species delimitations in this genus are still poorly resolved. The true diversity of *Opisthotropis* was underestimated, which is fatal for appropriate conservation of these habitat specialists. According to the integrative taxonomic approach, i.e., combining detailed morphological and molecular analyses, used in this study, the record of *O. maculosa* should be removed from the Chinese herpetofauna. So far, the true *O. maculosa* is restricted to northern Thailand, with only a single individual recorded. *Opisthotropis haihaensis* occurs in the mountain regions along the China-Vietnam border with only two female specimens recorded up to now, and *O. hungtai* is known only from the hilly regions between Guangxi and Guangdong of southern China. Extended surveys are urgently needed over the broad region from southern China to northern Thailand to investigate the distribution and the population status of these three species. Due to their beautiful color patterns, the snakes are in high demand in the animal trade market (Ziegler et al. 2019), and they must be evaluated for inclusion in one of the conservation categories in the IUCN Red List of Threatened Species.

The discovery of *Opisthotropis hungtai* sp. nov. brings the total number of species of *Opisthotropis* to 24. Nevertheless, with regard to recent phylogenetic results (Ren et al. 2017; Wang et al. 2017; Ziegler et al. 2019; this study), the relationships of clades within this genus still remain largely unresolved. As the mitochondrial CYTB gene is unable to generate significant support values, further work employing multilocus nuclear-gene and matrilineal mtDNA genealogy is recommended to decipher this puzzle. In addition, the similar appearance of *O. maculosa*, *O. haihaensis* and *O. hungtai*, together with their distant genetic divergence, indicates cryptic speciation in the genus *Opisthotropis*. The non-monophyletic relationships between *O. maculosa* and the clade composed of *O. hungtai* and *O. haihaensis* in our phylogenetic tree indicate that identical or similar phenotypes have evolved independently.

### Key to the species included in *Opisthotropis maculosa* sensu lato

**Table:** 

1	Prefrontal touching supraocular	***O. maculosa***
–	Prefrontal not touching supraocular	**2**
2	Supralabials eight, the second last one significantly enlarged; maxillary teeth 22–24	***O. haihaensis***
–	Supralabials seven, the second last one slightly enlarged; maxillary teeth 16–18	***O. hungtai***

## Supplementary Material

XML Treatment for Opisthotropis haihaensis

XML Treatment for Opisthotropis hungtai
